# Glycophenotypic Alterations Induced by *Pteridium aquilinum* in Mice Gastric Mucosa: Synergistic Effect with *Helicobacter pylori* Infection

**DOI:** 10.1371/journal.pone.0038353

**Published:** 2012-06-13

**Authors:** Joana Gomes, Ana Magalhães, Ana S. Carvalho, Gilberto E. Hernandez, Suzanne L. Papp, Steven R. Head, Valérie Michel, Leonor David, Fátima Gärtner, Eliette Touati, Celso A. Reis

**Affiliations:** 1 Institute of Molecular Pathology and Immunology of the University of Porto (IPATIMUP), Universidade do Porto, Porto, Portugal; 2 Institut Pasteur, Unité de Pathogenèse de Helicobacter, Paris, France; 3 The Scripps Research Institute, La Jolla, California, United States of America; 4 Faculdade de Medicina, Universidade do Porto, Porto, Portugal; 5 Instituto de Ciências Biomédicas Abel Salazar, Universidade do Porto, Porto, Portugal; Veterans Affairs Medical Center (111D), United States of America

## Abstract

The bracken fern *Pteridium aquilinum* is a plant known to be carcinogenic to animals. Epidemiological studies have shown an association between bracken fern exposure and gastric cancer development in humans. The biological effects of exposure to this plant within the gastric carcinogenesis process are not fully understood. In the present work, effects in the gastric mucosa of mice treated with *Pteridium aquilinum* were evaluated, as well as molecular mechanisms underlying the synergistic role with *Helicobacter pylori* infection. Our results showed that exposure to *Pteridium aquilinum* induces histomorphological modifications including increased expression of acidic glycoconjugates in the gastric mucosa. The transcriptome analysis of gastric mucosa showed that upon exposure to *Pteridium aquilinum* several glycosyltransferase genes were differently expressed, including *Galntl4*, *C1galt1* and *St3gal2*, that are mainly involved in the biosynthesis of simple mucin-type carbohydrate antigens. Concomitant treatment with *Pteridium aquilinum* and infection with *Helicobacter pylori* also resulted in differently expressed glycosyltransferase genes underlying the biosynthesis of terminal sialylated Lewis antigens, including Sialyl-Lewis^x^. These results disclose the molecular basis for the altered pattern of glycan structures observed in the mice gastric mucosa. The gene transcription alterations and the induced glycophenotypic changes observed in the gastric mucosa contribute for the understanding of the molecular mechanisms underlying the role of *Pteridium aquilinum* in the gastric carcinogenesis process.

## Introduction

The bracken fern *Pteridium aquilinum* (BF) is a common toxic plant that has high potential carcinogenic effects in animals and humans that consume BF or live in bracken-infested areas [1]. The BF, known to contain a toxin named ptaquiloside, is classified by the International Agency for Research on Cancer (IARC) as possibly carcinogenic to humans - group 2B [2], due to its carcinogenic and mutagenic effects [3]. Although clear carcinogenic effects have been shown in animals that consume BF [1], [3], the relationship between BF exposure and human health remains to be clarified. BF toxicity in humans has been associated with the consumption of the plant in some oriental cultures, and also with the indirect exposure to contaminated groundwater, drinking milk from cows fed with BF, and inhalation of bracken spores [4]–[6]. Epidemiologic evidences support association between BF exposure and gastric cancer development [7]–[9]. In agreement, we recently described the direct DNA damaging and mutagenic effects of BF and its toxin ptaquiloside in gastric epithelial cells and gastric mucosa of exposed mice [10].

Gastric cancer is the second leading cause of cancer-related death in the world [11]. The most common form of gastric cancer is the ultimate stage of a carcinogenic pathway that is initiated by the infection with *Helicobacter pylori* (*H. pylori*) [12], a type I human carcinogen according to IARC [13]. The gastric lesions associated with *H. pylori* infection can evolve over decades from chronic gastritis, gastric atrophy, intestinal metaplasia, and dysplasia to gastric carcinoma [14]. The development of this pathway leading to gastric carcinoma is a multifactorial process which depends on the interplay between several factors such as *H. pylori* virulence factors, host genetic polymorphisms and other environmental factors that dictate the clinical outcome of the disease.

Alterations in gastric glycophenotype are commonly observed during the gastric carcinogenic pathway and include increased expression of sialylated terminal structures, such as Sialyl-Lewis^x^ (SLe^x^) and Sialyl-Lewis^a^ (SLe^a^) [15]–[17], as well as aberrant expression of simple mucin-type carbohydrate antigens, as it is the case of Tn, Sialyl Tn (STn) and T antigens [18]–[20]. Some of these glycophenotypic alterations have been reported early in the process of *H. pylori* infection both in human gastric mucosa [15], [16] and experimentally infected animal models [21], [22]. Upon infection, *H. pylori* is able to modulate the expression of several host glycosylation-related genes, including the induction of a glycosyltransferase that results in increased SLe^x^ expression [23]. Modified glycosylation has been proposed to play a role in the development and progression of the disease [19], [24]. Particularly in gastric cancer, increased expression of sialylated antigens has been associated with a worst prognosis [25].

The goal of this study is to characterize the alterations induced by *Pteridium aquilinum* in the gastric mucosa and its possible synergistic effects with *H. pylori* infection. Histomorphological alterations and an altered glycophenotype were observed in the gastric mucosa of mice exposed to BF in the presence or absence of *H. pylori* infection. Furthermore, mice that were BF treated or concomitantly infected with *H. pylori* displayed different levels of glycosyltransferase genes expression as demonstrated using Glyco-gene Chip arrays. The identification of genes involved in the biosynthesis of carbohydrate antigens with a modified expression in gastric mucosa may contribute to a better understanding of the mechanisms involved in the development of gastric lesions associated to BF exposure and the contribution of *H. pylori* infection in this process.

## Results

### Phenotypic Alterations in Mice Gastric Mucosa in Response to *Pteridium aquilinum* Exposure and/or *H. pylori* Infection

**Figure 1:**
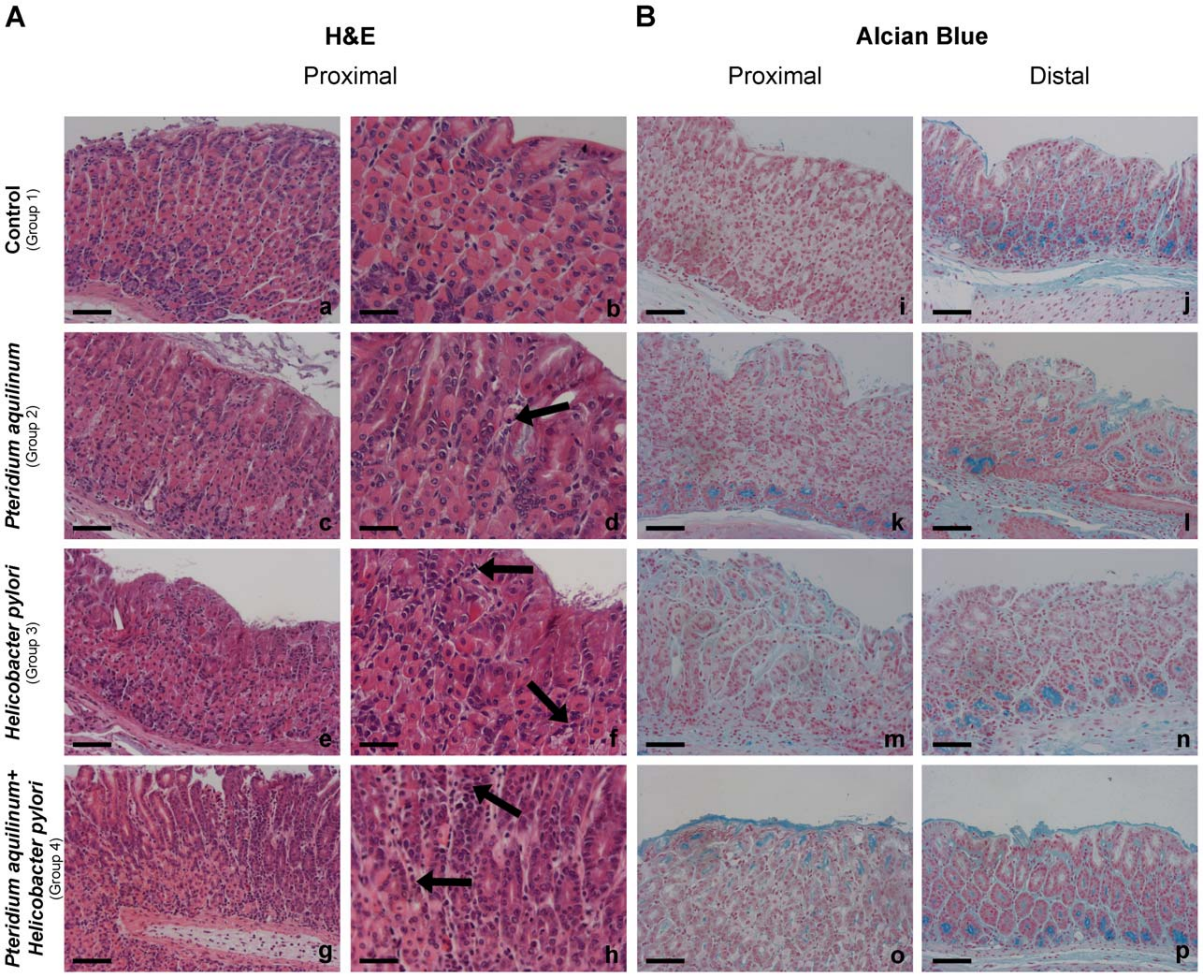
Histochemical characterization of mice gastric mucosa treated with *Pteridium aquilinum* and/or infected with *Helicobacter pylori.* Histochemical staining with hematoxylin and eosin (**Panel A**) and Alcian Blue (pH = 2,5) with nuclear red staining (**Panel B**) of mice gastric mucosa of: Control (Group 1) (a,b,i,j), *Pteridium aquilinum* treated (Group 2) (c,d,k,l), *Helicobacter pylori* infected (Group 3) (e,f,m,n) and both *Pteridium aquilinum* treated and *Helicobacter pylori* infected (Group 4) (g,h,o,p). Since the results observed at 4 and 7 weeks time points were identical, only a representative picture is included for each experimental condition. Bar = 40 µm (a,c,e,g and i-p) and bar = 20 µm (b,d,f,h). Arrows indicate inflammatory cells.

Histomorphological analysis of mice gastric mucosa stained with hematoxylin and eosin (H&E) (Figure 1, Panel A, a-h) showed various alterations in the BF treated (Group 2), *H. pylori* infected (Group 3) and BF treated and *H. pylori* infected (Group 4) mice groups when compared to the control (Group 1). The results observed at both time points (4 or 7 weeks) for each experimental condition were identical. Histological evaluation of inflammatory cells (Table S1) showed that gastric mucosa of BF treated (Group 2) mice displayed a mild inflammation when compared to control mice (Group1). Gastric mucosa of *H. pylori* infected (Group 3) mice displayed moderate inflammation in 5 of 8 evaluated animals while the others 3 animals showed mild inflammation. The concomitantly BF treated and *H. pylori* infected mice (Group 4) displayed severe inflammation with significant infiltrates of inflammatory cells in the gastric mucosa of all animals compared to control mice (Group1) in which the level of inflammatory components was classified as absent even though very few inflammatory cells could be observed (Figure 1, Panel A, a-h) (Table S1). BF treated mice also showed longer foveolar pits (Figure 1, Panel A, c-d) characterized by an increased number of cells in the same foveola. These structures frequently displayed increased acidic mucosubstances as demonstrated by Alcian Blue (pH 2.5) staining (Figure 1, Panel B, k-l). The gastric mucosa from the control animals (Group 1) showed minor acidic staining of few glands in the proximal region and a continuous moderate staining was observed in deep glands of the distal part of the stomach (Figure 1, Panel B, i-j). BF treated mice (Group 2) showed an increase of acidic staining in the foveolar epithelium and in the glands of both proximal and distal gastric mucosa (Figure 1, Panel B, k-l). *H. pylori* infected mice (Group 3) also showed enhanced acidic mucin staining in foveolar and deep glands (Figure 1, Panel B, m-n), which was even stronger in gastric mucosa of mice exposed to both BF treatment and *H. pylori* infection (Group 4) (Figure 1, Panel B, o-p).

**Figure 2:**
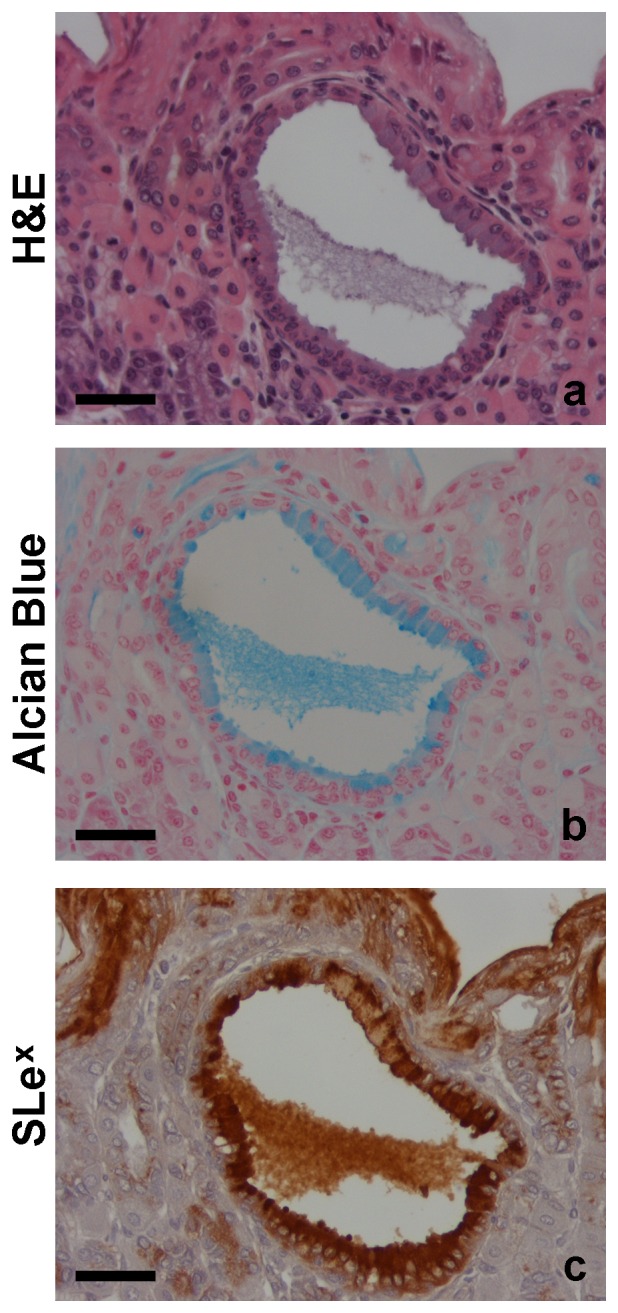
Characterization of cystically dilated gland of gastric mucosa of mouse exposed to *Pteridium aquilinum.* * Pteridium aquilinum* treated mice (Group 2) displaying a cystically dilated gland stained with hematoxylin and eosin (a), Alcian Blue (pH = 2,5) (b), and immunostained for Sialyl-Lewis x antigen (c). Bar = 20 µm.

In addition, cystically dilated glands were observed in three of the eight BF treated mice (Group 2) (Figure 2a). These structures showed acidic mucin expression as detected by Alcian Blue staining (Figure 2b).

*H. pylori* stomach colonization was also evaluated and no differences were observed when comparing *H. pylori* infected mice (Group 3) and *H. pylori* infected and BF treated (Group 4) (Figure S1). No colonization was observed in the gastric mucosa of non-infected mice (Groups 1 and 2).

### Deregulation of Glycosylation Induced by *Pteridium aquilinum* in the Gastric Mucosa of Mice

**Figure 3:**
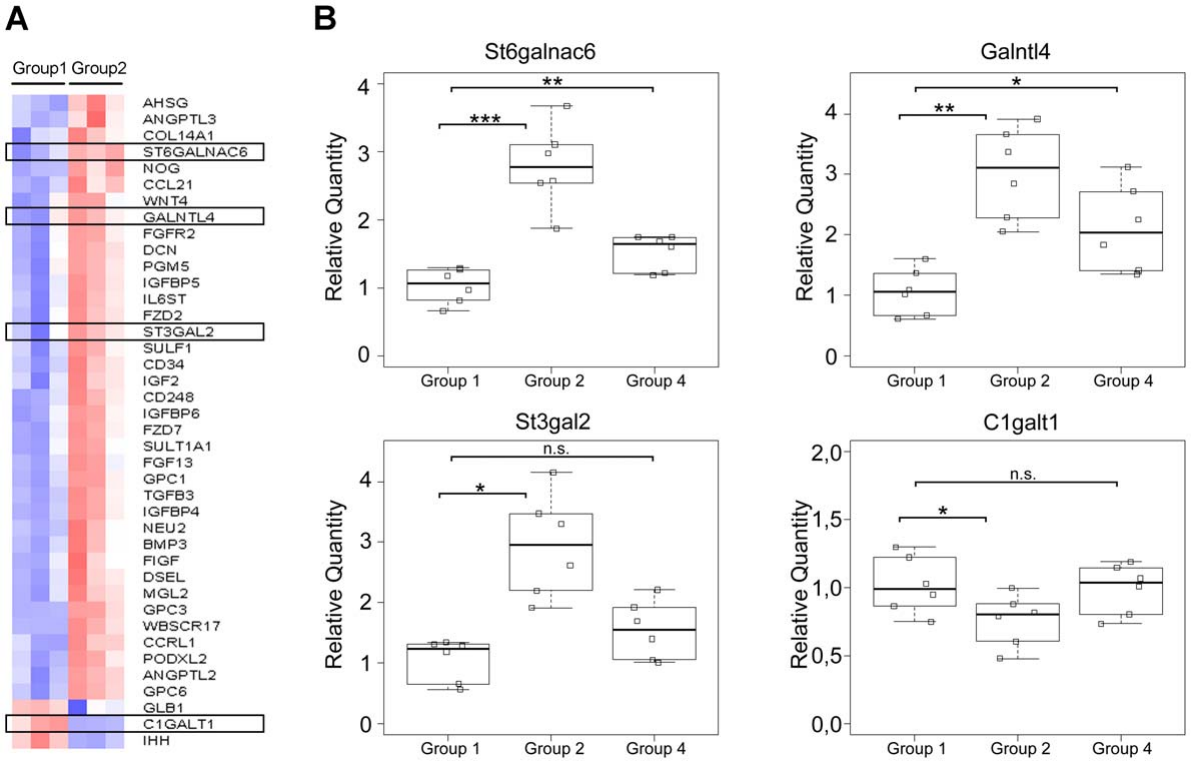
Glycosyltransferases expression analysis of gastric mucosa from *Pteridium aquilinum* exposed mice. Panel A – Heatmap of the Glyco-gene Chip array analysis obtained when comparing group 1 (control mice) with group 2 (mice treated with *Pteridium aquilinum*). Red and blue indicate increased and decreased expression relative to the mean transcript expression value, respectively. The transcripts identified as differentially expressed were those with adjusted p-value <0.1 and fold change >1.3. **Panel B –** Real-time PCR analysis for expression of *St6galnac6*, *Galntl4*, *St3gal2* and *C1galt1* in gastric mucosa from control (Group 1), *Pteridium aquilinum* treated (Group 2) and concomitantly *Pteridium aquilinum* treated and *Helicobacter pylori* infected (Group 4) mice. The gene used as reference was *Hprt1*. Significance was evaluated using the Shapiro-Wilk test. n.s. not significant p<0.05(*); p<0.01(**); p<0.005 (***).

In order to identify the genes underlying the modified glycosylation pattern observed upon BF treatment, a transcriptome analysis was performed using the Glyco-gene Chip array. The comparison of gene expression from BF treated mice (Group 2) with control mice (Group 1) identified 44 differentially expressed transcripts with a fold change >1.3 (p-value <0.1). The heatmap for this analysis was generated showing the mean-scaled expression for these comparisons (Figure 3, Panel A). The genes with an altered expression in response to BF treatment included glycosyltransferases involved in O-glycan biosynthesis and controlling the simple mucin-type carbohydrate antigen expression, often deregulated during gastric carcinogenesis. *St6galnac6* (NM_016973.2), *Galntl4* (NM_173739.3) and *St3gal2* (uc009nlk.1) genes showed an increased expression whereas the expression of *C1galt1* (NM_052993.2) decreased (Table S2).

These altered gene expression levels observed by microarrays were confirmed by qRT-PCR (Figure 3, Panel B). *St6galnac6, Galnactl4,* and *St3gal2* showed a 2.79 (p-value 0.0004), 3.02 (p-value 0.0006) and 2.94 (p-value 0.002) fold increased expression, respectively, in BF treated mice (Group 2), in comparison with the control group (Group 1). In addition, *C1galt1* reduced expression was also confirmed by qRT-PCR showing a 1.32 fold reduction (p-value 0.05) (Figure 3, Panel B), when compared with non-treated mice (Group 1).

**Figure 4:**
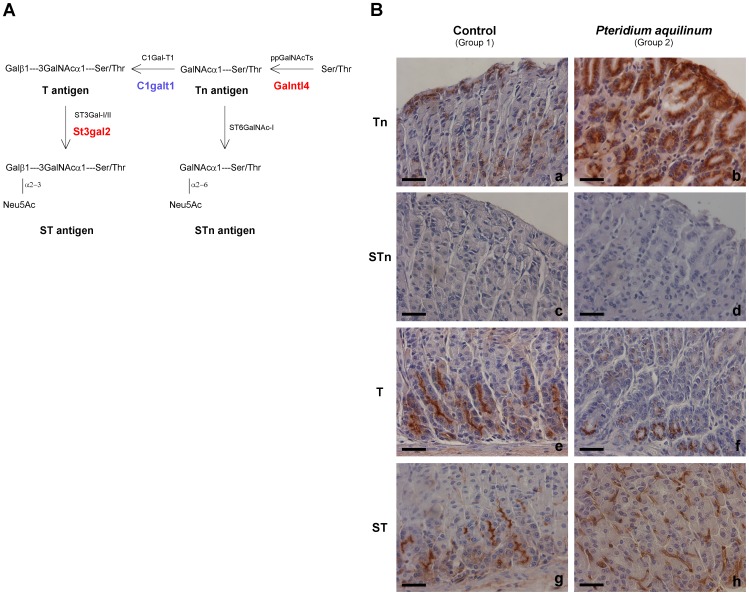
Alterations in simple mucin-type carbohydrate antigens in gastric mucosa from mice exposed to *Pteridium aquilinum.* Panel A – Schematic representation of the biosynthesis of simple mucin-type carbohydrate antigens. The enzymes or family of enzymes responsible for each step of the biosynthesis are indicated. The enzymes identified as showing different expression levels in *Pteridium aquilinum* treated mice (Group 2) are highlighted in red (increased expression) and blue (decreased expression). **Panel B** – Immunohistochemistry of simple mucin-type carbohydrate antigens, Tn (a,b), STn (c,d), T (e,f) and ST (g,h) in gastric mucosa of control (Group 1) (a,c,e,g) and *Pteridium aquilinum* treated (Group 2) (b,d,f,h) mice. Bar = 20 µm.

The glycosyltransferases Galnactl4, C1galt1 and St3gal2 participate in the initial steps of protein O-glycosylation and are involved in the biosynthesis of simple mucin-type carbohydrate antigens (Figure 4, Panel A). Detailed analysis of simple mucin-type carbohydrate antigens expression in gastric mucosa of control (Group 1) and BF treated mice (Group 2) was evaluated by immunohistochemistry (Figure 4, Panel B and Table 1). Increased Tn antigen expression with a stronger positive staining was observed in BF treated (5/8) compared to control mice (0/7) (p-value 0,026) (Figure 4, Panel B, a-b) (Table 1). Both control (Group 1) and BF treated mice (Group 2) showed no STn antigen expression (Figure 4, Panel B, c-d) (Table 1). Expression of T antigen was observed in both BF treated and control, however a more limited staining was observed in BF treated mice (Group 2) (Figure 4, Panel B, e-f) (Table 1). Sialylation of T was evaluated comparing positivity to T antigen before and after neuraminidase treatment. ST expression was considered as the surplus of staining when compared to T antigen. BF treated mice (Group 2) showed an increase of ST expression (6/8) when compared to gastric mucosa of control mice (0/7) (p-value 0,007) (Group 1) (Figure 4, Panel B, g-h) (Table 1).

**Table 1:** Expression of carbohydrate antigens a in control, *Helicobacter pylori* infected and *Pteridium aquilinum* treated with or without infection mice.

		**Tn**				**STn**				**T**				**ST**					**SLe** a				**SLe^x^**			
		**-**	**+**	**++**	**p value** b	**-**	**+**	**++**	**p value** b	**-**	**+**	**++**	**p value** b	**-**	**+**	**++**	**p value** b		**-**	**+**	**++**	**p value** b	**-**	**+**	**++**	**p value** b
**Control**	**n = 7**	0	7	0	-	7	0	0	-	0	7	0	-	7	0	0	-	**n = 7**	7	0	0	-	0	7	0	-
***Pteridium*** ***aquilinum***	**n = 8**	0	3	5	0,026	8	0	0	n.a.	0	8	0	n.a.	2	6	0	0,007	**n = 8**	8	0	0	n.a.	0	2	6	0,007
***Helicobacter*** ***pylori***	**n = 5**	0	4	1	0,417	5	0	0	n.a.	0	5	0	n.a.	5	0	0	n.a.	**n = 7**	7	0	0	n.a.	0	4	3	0,192
***Pteridium*** ***aquilinum*** ** + ** ***Helicobacter*** ***pylori***	**n = 5**	0	0	5	0,001	5	0	0	n.a.	0	5	0	n.a.	2	3	0	0,045	**n = 7**	7	0	0	n.a.	0	0	7	0,001

a Staining was graded in three categories: (-) negative, (+) positive, and (+ +) strongly positive.

b Fisher’s exact test (p-value); n.a. – not applicable.

### Glycosylation Alterations in Mice Gastric Mucosa upon *Pteridium aquilinum* Treatment and Concomitant *H. pylori* Infection

**Figure 5:**
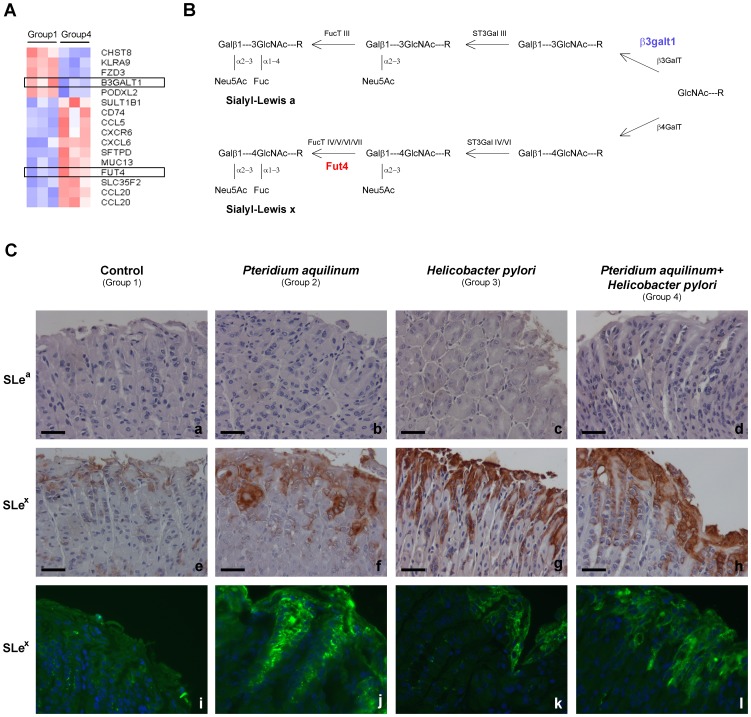
Glycosylation alterations in mice gastric mucosa upon *Pteridium aquilinum* treatment and concomitant *Helicobacter pylori* infection. **Panel A –** Heatmap of the Glyco-gene Chip array analysis obtained when comparing group 1 (control) with group 4 (treated with *Pteridium aquilinum* and *Helicobacter pylori* infected). Red indicates increased and blue indicates decreased expression relative to the mean transcript expression value. The transcripts identified as differentially expressed were those with adjusted p-value <0.1 and fold change >1.3. **Panel B** – Schematic representation of the biosynthesis of sialylated terminal structures. The enzymes or family of enzymes responsible for each step of the biosynthesis are indicated. The enzymes identified as showing different expression levels in *Pteridium aquilinum* treated and *Helicobacter pylori* infected mice (Group 4) are highlighted in red (increased expression) and blue (decreased expression). **Panel C** – Immunohistochemistry for evaluation of expression of Sialyl-Lewis a (SLe^a^) antigen (a–d) and for Sialyl-Lewis x (SLe^x^) (e–h). Additional immunofluorescence evaluation performed for SLe^x^ (i–l). Bar = 20 µm.

The Glyco-gene Chip array analysis of the samples from BF treated mice and *H. pylori* infected (Group 4) compared to control mice (Group 1) identified 16 differentially expressed transcripts with a fold change >1.3 (p-value <0.1). The heatmap for this analysis was generated showing the mean-scaled expression for these comparisons (Figure 5, Panel A). The genes with an altered expression included *B3galt1* (NM_020283.2) encoding for a member of the ß1–3 galactosyltransferase family which is involved in the extension of oligosaccharide chains [26] and *Fut4* a fucosyltransferase (NM_010242.3) that participates in the biosynthesis of terminal Lewis antigen structures [27] (Figure 5, Panel B) (Table S3). The Glyco-gene Chip array analysis showed no differences when comparing *H. pylori* infected mice (Group 3) with the control mice (Group 1).

When overlapping the transcripts differentially expressed in groups 2 and 4 only one transcript in common was observed, the podocalyxin-like 2 (*Podxl2*) (NM_176973.3), a selectin ligand – adhesion molecule [28]. We also analyzed by qRT-PCR the expression of the genes that showed a higher fold variation upon BF treatment (Group 2) in the samples from group 4. This analysis showed an increased expression for *St6galnac6* (1.5 fold higher, p-value 0.007) and *Galntl4* (2.1 fold higher, p-value 0.01) in group 4 compared to group 1, as observed for the group 2 (Figure 3, Panel B). No significant alterations were seen for the expression of *St3gal2* and *C1galt1* in group 4 (Figure 3, Panel B).

Immunohistochemical evaluation of simple mucin-type carbohydrate antigens expression was done in gastric mucosa of *H. pylori* infected mice (Group 3) and mice concomitantly treated with BF and infected with *H. pylori* (Group 4) (Table 1). A strong increase of Tn antigen expression was observed in concomitantly BF treated and *H. pylori* infected (5/5) (p-value 0,001) when compared to control group while no significant difference was observed in *H. pylori* infected mice (1/5) (Table 1). On the other hand, no STn antigen expression was detected in both groups (Table 1). Despite both groups are positive (5/5) for T antigen only concomitantly BF treated and *H. pylori* infected mice (Group 4) showed ST expression in some mice gastric mucosa (3/5) (p-value 0,045) (Table 1).

The analysis of the expression of the sialylated antigens, SLe^x^ and SLe^a^, was also performed by immunohistochemistry (Figure 5, Panel C, a-h) (Table 1). SLe^x^ overexpression was observed in superficial foveolar glands of BF treated mice (6/8) (p-value 0,007) (Group 2) as compared to control mice (Group 1). Mice infected with *H. pylori* (Group 3) also showed a trend towards an increased SLe^x^ staining (3/7) although not reaching statistical significance. Furthermore, gastric mucosa from mice concomitantly treated with BF and infected with *H. pylori* (Group 4) presented a strong SLe^x^ expression in all animals evaluated (7/7) (p-value 0,001) (Figure 5, Panel C, e-h) (Table 1).

Immunofluorescence analysis showed similar results for SLe^x^ detection (Figure 5, Panel C, i-l). Cystically dilated glands observed in the histomorphological analysis of BF treated mice (Group 2) with a strong positive Alcian blue acidic staining also showed a high positive SLe^x^ staining (Figure 2c).

No expression of the SLe^a^ antigen was detected in the gastric mucosa of any mice from all the evaluated groups (Figure 5, Panel C, a-d).

## Discussion

In the present study, we have analyzed, in a mouse model, the ability of BF to induce glycophenotypic alterations in the gastric mucosa that could be involved in the gastric carcinogenesis process. BF has been previously demonstrated to induce acute and chronic toxic effects leading to cancer development on livestock and in animal models [1], [29]. In addition, epidemiological studies have shown higher incidence of gastric cancer in humans directly or indirectly exposed to BF [1]. The role of BF and its ptaquiloside toxin in the gastric carcinogenesis process is also supported by the induction of DNA damaging effects in human gastric epithelial cells and in mouse gastric mucosa exposed to these toxic agents [10].

Our results showed that gastric mucosa of C57Bl/6 mice treated with BF (Group 2) displays morphological alterations characterized by longer gastric foveolar pits, increased number of inflammatory cells, and development of cystically dilated glands. These histomorphological alterations are in agreement with the increased gastric epithelial cells proliferation previously observed upon BF treatment in the gastric mucosa of mice [10]. In addition, our results showed an increased expression of acidic mucosubstances in the foveolar superficial epithelium and in both proximal and distal glands of the gastric mucosa. Alterations in the acidic characteristics of the gastric mucosa have been previously reported during gastric carcinogenesis [30], [31]. These alterations are due to modification of the glycan structures on glycoconjugates, such as glycoproteins and glycolipids, that are produced by gastric epithelial cells [17]–[19].

The pattern of glycosylation observed in glycoconjugates produced by gastric epithelial cells depends on the coordinated action of various glycosyltranferases [20]. The transcriptome analysis of the mice gastric mucosa using the Glyco-gene Chip array showed that BF induced differently expressed glycosyltransferase genes, including *Galntl4*, *C1galt1* and *St3gal2*, mainly involved in the biosynthesis of simple mucin-type carbohydrate antigens. GalNAc-TL4 is a member of the UDP-GalNAc-polypeptide *N*-acetylgalactosaminyl-tranferases (ppGalNAc-Ts) family participating in the synthesis of the Tn antigen [32], [33]. These ppGalNAc-Ts have shown to display different kinetic and acceptor substrate specificities as well as tissue expression [34]–[36]. GalNAc-TL4 is also known as ppGalNAc-T18 [32], [37]. The increased levels of *Galntl4* expression and decreased expression of *C1galt1* could explain the different glycophenotype observed upon BF treatment, including the increased detection of the Tn antigen by immunohistochemistry. Furthermore, our results also showed no alterations in the St6galnac1 expression, nor in the STn antigen expression.

In normal cells, the Tn antigen is further processed by the C1Gal-T1 enzyme that adds Gal to GalNAc, leading to the synthesis of core 1 structure (T antigen) [38]. The downregulation of C1galt1 observed in BF treated mice (Group 2) explains the weaker immunodetection of the T antigen in the gastric mucosa of these mice. T antigen can be sialylated by sialyltransferases, ST3Gal-I [39] or ST3Gal-II [40], leading to the biosynthesis of ST antigen [41]. Furthermore, the increased expression of *St3gal2* provides the molecular evidence supporting that most core 1 structures are α2,3 sialylated leading to the ST antigen biosynthesis, as observed by the T antigen detection before and after neuraminidase treatment. This increased detection of sialylated glycoepitopes is in agreement with the increased histochemical detection of acidic glycoconjugates observed. Alteration in expression of sialyltransferases, enzymes that catalyze the transfer of sialic acid to the terminal position of the carbohydrate group of glycoproteins and glycolipids [42], [43] has been described in various diseases models, including cancer in which it can correlate with cells invasion capacity [41]. St3gal2 has also been shown to participate in the biosynthesis of gangliosides [40].

In addition, another sialyltransferase, St6galnac6 which is specific for glycolipid acceptors, catalyzing the transfer of sialic acid in an α2,6 linkage onto GalNAc in GT1b and GD1a, leads to the synthesis of gangliosides GQ1bα and GT1aα [44], [45]. These glycolipids have been shown to play an important role in different biological events such as cell-cell interaction, cell migration, adhesion, and metastization [43]. The St6galnac6 enzyme also showed to be upregulated in gastric mucosa of BF treated mice (Group 2). This data is in agreement with the increased inflammation observed in the gastric mucosa of these mice. These features corroborate previous studies showing that changes in α2,3-, α2,6-, and α2,8-sialic acid glycotopes can be induced during inflammatory processes [23], [46].

Alterations in host gastric mucosa glycosylation phenotype have been reported early during *H. pylori* infection [15], [16]. *H. pylori* is a bacterium that infects the gastric mucosa being responsible for most gastric diseases including the development of gastric cancer for which it constitutes a major risk factor [14]. The interplay between host genetic susceptibility factors, environmental factors, and *H. pylori* infection underlies the progress in the carcinogenesis process [47]. In the present study, we have further evaluated a possible synergistic effect of *H. pylori* infection and BF exposure in the gastric carcinogenesis process.

Our results showed that gastric mucosa of C57Bl/6 mice infected with *H. pylori* displayed an increased number of inflammatory cells in the gastric mucosa independently of BF treatment. In addition, concomitant BF treatment and *H.pylori* infection (Group 4) enhanced acidic mucin staining in foveolar and deep glands of mice gastric mucosa when compared with the control ones (Group 1). No altered levels in the expression of the enzymes involved in the biosynthesis of simple-mucin type antigens were observed in the microarray for this group. However, real-time PCR analysis showed moderately increased levels of expression of *St6galnac6* and *Galntl4* genes in the gastric mucosa of these mice (Group 4). These differences may be due to the different sensitivity of the two methodological approaches.

Alterations in glycosylation observed in the human gastric mucosa of infected individuals included the expression of sialylated terminal structures, such as SLe^x^ and SLe^a^ [15], [16] due to modified expression of glycosyltransferases [23], generating the biosynthesis of additional glycan receptors for *H. pylori* adhesins [15]. Glycosyltransferases, like galactosyltransferases, sialyltransferases and fucosyltransferases, are involved in such biosynthesis of terminal Lewis antigens [20], [48].

In our Glyco-gene Chip array analysis we did not observed significant alterations in *H. pylori* infected group (Group 3). However, the concomitant BF treatment and *H. pylori* infection (Group 4) induced significant changes including down-regulation in *B3galt1* and an up-regulation in *Fut4*. The enzyme β3galt1 belongs to the β3-galactosyltransferases family that catalyses the formation of type 1 structure Galβ1-3GlcNac [26], [49] that serves as acceptor for the biosynthesis of type 1 Lewis antigens, including SLe^a^. On the other hand, another family of enzymes, the β4-galactosyltransferases synthesize type 2 carbohydrate chains [50], which are the acceptors for sialyltransferases and fucosyltransferases, like Fut4 [27] that participates in the biosynthesis of SLe^x^. No SLe^a^ immunostaining was observed in the gastric mucosa of all different groups of mice, as previously described [51].

Our results showed an increased SLe^x^ expression when comparing the control mice (Group 1) with BF treated mice with (Group 4) or without (Group 2) *H. pylori* infection. The stronger expression of SLe^x^ observed in gastric mucosa of mice concomitantly treated with BF and infected with *H. pylori* (Group 4) corroborates the importance previously attributed to SLe^x^ during chronic inflammation of gastric mucosa. The biological relevance of the SLe^x^ expression may be partly due to the role that this glycan receptor plays in the context of *H. pylori* binding through its sialic-acid binding adhesin, SabA, to gastric cells [15]. Previous studies have shown that overexpression of β3GnT5, a GlcNAc transferase essential for the first step of biosynthesis of Lewis antigens, was observed in human gastric cells upon *H. pylori* infection, leading to increased SLe^x^ expression and increased SabA mediated bacterial adhesion [23]. In the present study, the promotion of gastric mucosa inflammation together with an increased expression of SLe^x^ observed in the mice gastric epithelium, suggest that the expression of ligands for bacterial adhesins, as SabA, could be modulated upon BF exposure and *H. pylori* infection. Overall, these studies support the importance of glycan receptors as mediators in the adhesion of the gastric pathogen *H. pylori* [52]. Although the present study used a limited number of mice within each study group, our results clearly demonstrate significant glycophenotypic alterations induced by *H. pylori* and BF.

**Table 2:** Specificity and dilution of monoclonal antibodies.

Specificity	mAb	Dilution	References
**Tn** (GalNAcαThr/Ser)	1E3	1∶5	Clausen and Hakomori, unpublished
**Sialyl-Tn** (NeuAcα2-6GalNAcαThr/Ser)	TKH2	1∶100	[59]
**T** (Galβ1-3 GalNAcαThr/Ser)	3C9 a	1∶25	[60]
**Sialyl-Lewis^x^** (NeuAcα2-3Galβ1-4(Fucα1-3)GlcNAc-R)	KM93	1∶40	Calbiochem
**Sialyl-Lewis** a (NeuAcα2-3Galβ1-3(Fucα1-4)GlcNAc-R)	Ca19.9	1∶500	Santa Cruz

a Sialyl-T antigen was detected using the anti-T antibody after neuraminidase treatment as described in Material and Methods.

In agreement with our data, it has been shown that *H. pylori* infection induces changes in glycosylation in the gastric mucosa of animal models such as Rhesus monkeys and Mongolian gerbils [22], [53]. Furthermore, synergistic effects of *H. pylori* infection and the ingestion of a carcinogen present in the diet have been observed in the Rhesus monkey model, including histomorphologic alterations and modification of cancer-associated gene expression [54].

While our observations demonstrate the glycophenotypic alterations of host cells in response to BF exposure and *H. pylori* infection, other studies have focused on *H. pylori* glycan-gene expression. A recent study pointed out the importance of two *H. pylori* genes, *jhp0562*, which encodes a glycosyltransferases involved in the synthesis of the bacterial lipopolysacharide and the immediately upstream gene, *jhp0563* that encodes a β1,3 galactosyltransferase involved in bacterial Lewis antigen synthesis. These genes are associated with different gastric pathologies allowing the discrimination between peptic ulcers and gastritis in some populations [55]. These studies further demonstrate the important role that glycosylation plays in the modulation of host-pathogen interactions.

In conclusion, our results show that the bracken fern *Pteridium aquilinum* is able to induce various histomorphologic and molecular changes in the gastric mucosa. These modifications include major alterations at the transcriptional level of several genes, such as glycosyltransferases involved in the biosynthesis of simple mucin type carbohydrate antigens. Furthermore, the concomitant exposure to BF and *H. pylori* infection also induced alterations at the transcriptional level of glycosyltransferases participating in the biosynthesis of terminal glycan Lewis antigens. These gene transcription modifications underly the increased acidic glycoconjugate phenotype observed in BF exposed in gastric mucosa in the context of *H. pylori* infection. Our results contribute for understanding the molecular mechanisms underlying the role of BF pathogenic consequences in gastric mucosa and its synergistic effects with *H. pylori* infection.

**Table 3:** Primer sequences and Real-time PCR conditions for the analysis of gene expression in mice gastric mucosa.

Gene	Primers Real-time PCR	Amplicon (bp)		PCR
***St6galnac6***	*for* 5′- GCAACAAGACGCTGCCGTCC - 3′	107		
	*rev* 5′- GGTGCACTCAGCCCGCTCAA - 3′			
***Galntl4***	*for* 5′- CCCTCATTGGCTGTTTCATT - 3′	106		
	*rev* 5′ - CTGATCCCAAGCTCCACATT - 3′			10 min, 95°C
***St3gal2***	*for* 5′- ATGGCTACCTTGCCCTACCT - 3′	203	45 times	15 s, 95°C
	*rev* 5′- GTCCAGACGGGTGAGATGTT - 3′			60 s, 60°C
***C1galt1***	*for* 5′- TGCAGATTCCAGCCAACATAAAGATGA - 3′	130		
	*rev* 5′- AGCTTTGACATGTTTGGCCTTTTTCTC - 3′			
***Hprt1***	*for* 5′- AGCTACTGTAATGATCAGTCAACG - 3′	198		
	*rev* 5′- AGAGGTCCTTTTCACCAGCA - 3′			

## Materials and Methods

### Animal Experiments and Tissue Samples

Four groups of six-week-old specific pathogen-free C57Bl/6 male mice (Charles River Laboratories) were used in this study according with the Specific Guide for the Care and the Use of Laboratory Animals of the Institut Pasteur, the European Directive (2010/63/UE) and the corresponding French law on animal experimentation. The protocol was also approved by the Committee of Central Animal Facility Board of the Institut Pasteur.

Each group consisted in 8 animals. Mice from the group 1 (control) received drinking water during all the experiment. The group 2 was exposed to BF aqueous extracts, obtained as previously described [56]. In this group mice received, during the third and fourth weeks of the experiment, a drinking water supplemented with BF extracts (250 mg BF/ml) as already reported [10]. In the group 3, mice were orogastrically infected with *H. pylori* strain SS1 (100 µl of 10^7^ bacteria), twice during the first week. In the group 4, mice were orogastrically infected with *H. pylori* strain SS1 (100 µl of 10^7^ bacteria) and treated with BF extracts in the same conditions described for group 2. From each experimental group, 4 mice were sacrificed after 4 and 7 weeks from the beginning of the experiment and their stomach collected. *H. pylori* gastric colonization was quantified in the gastric mucosa as previously described [57].

### Histochemistry and Immunohistochemistry

Mice gastric mucosa fragments including antrum and corpus were formalin-fixed and embedded in paraffin wax before serial sections were cut.

H&E staining was done to observe morphological alterations in gastric mucosa. Alcian blue (pH 2.5) (Merck) was used to detect the presence of acidic mucins and nuclear red 1% used to stain the nuclei.

A semi-quantitative evaluation of the inflammatory cells was done in all mice gastric mucosa according to the Sidney Classification System [58] and graded on a scale of absent, mild, moderate and severe infiltrate (Table S1). A representative figure of each group illustrates the results observed (Figure 1).

Immunohistochemical analysis of carbohydrate antigens was performed according to the standard avidin-biotin-complex staining method using the monoclonal antibodies described in table 2 [59], [60]. Analysis was performed in samples from both time points. We used 7 gastric mucosa samples from control mice (Group 1) and 8 samples from BF treated mice (Group 2) for all the antigens evaluated. We used 5 gastric mucosa from *H. pylori* infected (Group 3) and concomitantly BF treated and *H. pylori* infected mice (Group 4) for Tn, STn, T and ST antigen evaluation while 7 gastric mucosa were used to SLe^a^ and SLe^x^ antigens detection (Table S1).

Briefly, tissue samples were deparaffinated, rehydrated and incubated at 37°C with neuraminidase from *Clostridium perfringes* type VI (Sigma) diluted in 0.1 M sodium acetate buffer (pH 5.5) followed by washes in ice-cold when needed (recognition of ST using 3C9 MAb). Sections were treated with 0.3% hydrogen peroxide in methanol to block endogenous peroxidase, incubated with normal rabbit serum diluted 1∶5 in PBS with 10% of BSA and then incubated overnight at 4°C with the respective monoclonal primary antibody (Table 2) [59], [60]. The slides were then washed in PBS and incubated with biotinylated rabbit anti-mouse secondary antibody (DakoCytomation) diluted 1∶200 in PBS with 5% of BSA prior to the incubation with avidin-biotin peroxidase complex (Vectastain Elite ABS kit). Sections were stained with 3,3′-Diaminobenzidine tetrahydrochloride (Sigma) in a buffer containing 0.1% hydrogen peroxide, counter-stained with Mayer’s hematoxylin, dehydrated and mounted. Negative controls were performed by replacing primary antibody with PBS. Staining was graded as -, negative; +, positive; + +, strongly positive.

Statistical analysis was performed using the Chi Square and Fisher’s exact tests with Statview 5.0 software. Differences were considered statistically significant at p<0.05.

### Immunofluorescence Assay

Mice gastric tissues were deparaffinated, rehydrated and incubated with rabbit non-immune serum (DakoCytomation) diluted 1∶5 in PBS with 10% of BSA. Sections were incubated overnight at 4°C with SLe^x^ antibody (KM93, Calbiochem) diluted in PBS containing 5% of BSA. Sections were then washed in PBS and incubated with FITC-conjugated rabbit anti-mouse immunoglobulin (DakoCytomation) diluted 1∶70 in PBS with 5% of BSA. Sections were washed in PBS and incubated with DAPI 100 µg/µL (Sigma). Samples were washed in PBS and mounted in VectaShield (Vector Laboratories).

### Glyco-gene Chip Arrays and Analysis of Data

Total RNA from gastric mucosa of 3 mice from each experimental condition (biological replicates) sacrificed after 7 weeks from the beginning of the experiment, was extracted using the RNeasy Plus Mini kit (QIAGEN) according to the manufacturer’s protocol. RNA yield and quality were determined using NanoDrop ND-1000 spectrophotometer (THERMO Scientific).

A total of 12 samples were hybridized on the GlycoV4 oligonucleotide arrays specially developed for the Consortium for Functional Glycomics - CFG, using the Affymetrix technology (Affymetrix, USA) and containing probes that allow to detect the expression of 1200 mouse glyco-related transcripts [61]. The following classes of genes are represented on the GLYCOv4 Gene Chip: Glycosyltransferases, Glycan-binding proteins (GBPs), including C-type lectins, galectins and siglecs, Glycan degradation proteins, Intercellular protein transport proteins, Notch pathway proteins, Nucleotide sugar synthesis and transporter proteins, N-glycan biosynthesis-related proteins, Adhesion molecules, Interleukins and receptors, Mucins, Growth factors and receptors, Cytokines, Chemokines, Conserved oligomeric Golgi (COG) complex proteins and other miscellaneous proteins of interest. The complete list of genes present on the GLYCOv4 Gene Chip array is available at http://www.functionalglycomics.org/static/consortium/resources/resourcecoree.shtml.

Data normalization was performed using RMA Express 1.0 with quantile normalization, median polish and background adjustment [62], [63].

The Limma package in the R software [64] was used to find transcripts with differential expression. The fold changes and standard errors were estimated by fitting a linear model for each gene and empirical Bayes smoothing was applied to the standard errors. Results are presented between two or more experimental conditions as a fold change in expression level, the moderated t-statistic, the p-value, and the adjusted p-value. The adjusted p-value is the p-value adjusted for multiple testing using the Benjamini and Hochberg’s method [65], to control the false discovery rate of 0.1 or less. The transcripts identified as differentially expressed were those with adjusted p-value <0.1 and fold change >1.3.

Heatmaps were generated with dChip program comparing group 1 (control) with group 2 (treated with BF) and comparing group 1 (control) with group 4 (infected with *H. pylori* and treated with BF). No heatmap was generated for group 3 (infected with *H. pylori*) since no difference was observed when comparing to control group 1. Red and blue indicates increased and decreased expression relative to the mean transcript expression value, respectively.

### cDNA Synthesis

First strand cDNA was produced from 2.5 µg of total RNA from the same samples evaluated in the Glyco-gene Chip array, using Superscript II Reverse Transcriptase kit (Invitrogen) in accordance with the manufacturer’s instructions. The reaction mixture was incubated at 25°C for 10 min, 42°C for 50 min and 15 min at 72°C.

### Relative Quantitative Real-time PCR and Statistical Analysis

Relative Quantitative real-time PCR was performed in the groups showing gene expression alterations detected in the glycogene chip array.

qRT-PCR was done using the SYBR®Green chemistry in a 7500 Fast Real Time PCR System (Applied Biosytems, Foster City, CA, USA). The reaction mixture contained 2 µl cDNA (diluted 1∶2 in water), 10.0 µl Power SYBR®Green PCR Master Mix (2x) (Applied Biosystems), and 0.48 µl of each 10 µM primer (Sigma Genosys) in a final reaction volume of 20 µl. Primers melting temperature, amplicon length and sequence are resumed in Table 3. The cycling conditions were as follows: denaturation at 95°C for 10 min, followed by 45 cycles of 95°C for 15 s and 60°C for 60 s, and a final step (95°C for 15 s, 60°C for 60 s, 95°C for 30 s, 60°C for 15 s to create a dissociation curve in order to assure assay specificity. Calculations were made using the comparative C_T_ method (2^-ΔΔCT^). *Hprt1* was used as an endogenous control gene for PCR normalization to the amount of RNA added to the reverse transcription reactions, and the biological group 1 as the calibrator. Triplicates of each biological group were analyzed twice independently.

Normality of the data was in all cases confirmed by Shapiro-Wilk test [66]. Welch none paired two sample t-test was used to calculate the p-values for each gene, between biological groups with the null hypothesis of equal means.

## Supporting Information

Figure S1 **Evaluation of** ***Helicobacter pylori*** **colonization in gastric mucosa of infected mice (Group 3) and infected and** ***Pteridium aquilinum*** **treated mice (Group 4) at 4 and 7 weeks.** Colonization represented by the number of CFU/g of mice stomach tissue. Each point represents one mouse and the mean value is also shown.
(TIF)

Table S1 **Characterization of inflammation and immunohistochemistry analysis of carbohydrate antigens expression in the gastric mucosa of all mice evaluated in each experimental group.**
(PDF)

Table S2 **Significantly altered genes in** ***Pteridium aquilinum*** **treated gastric mucosa (Group 2) in comparison with control (Group 1) from the Glyco-gene Chip array analysis.**
(PDF)

Table S3 **Significantly altered genes in** ***Pteridium aquilinum*** **treated and** ***Helicobacter pylori*** **infected gastric mucosa (Group 4) in comparison with control (Group 1) from the Glyco-gene Chip array analysis.**
(PDF)
