# Supplementary figures and images for: Glycophenotypic Alterations Induced by Pteridium aquilinum in Mice Gastric Mucosa: Synergistic Effect with Helicobacter pylori Infection
Source: PLoS One. 2012 Jun 13;7(6):e38353. doi: 10.1371/journal.pone.0038353 (PMC3374793; doi:10.1371/journal.pone.0038353)

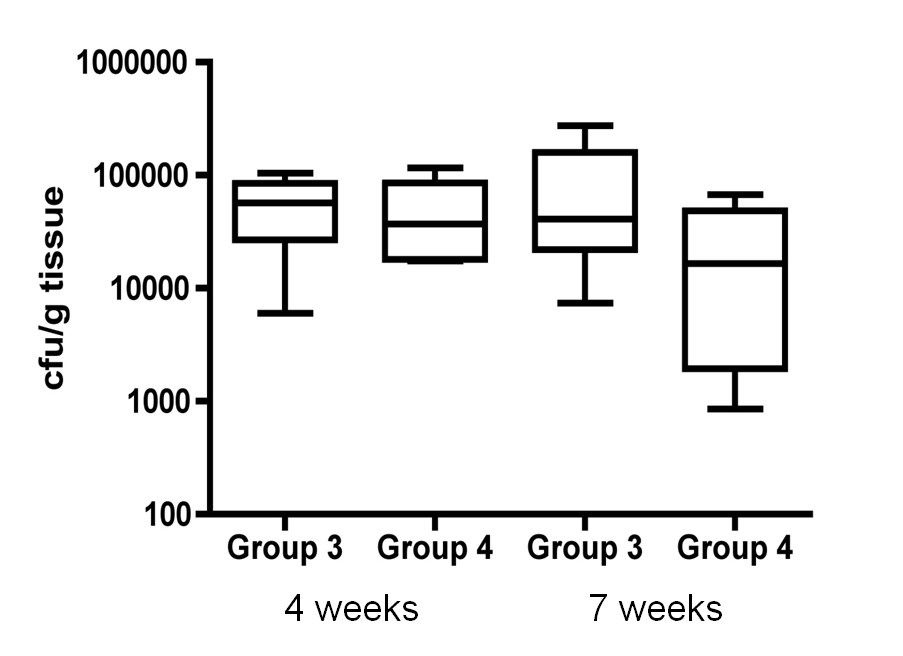

Supplement: Figure S1 — Evaluation of Helicobacter pylori colonization in gastric mucosa of infected mice (Group 3) and infected and Pteridium aquilinum treated mice (Group 4) at 4 and 7 weeks. Colonization represented by the number of CFU/g of mice stomach tissue. Each point represents one mouse and the mean value is also shown. (TIF) [file pone.0038353.s001.tif]
