# Supplementary material for: Glycophenotypic Alterations Induced by Pteridium aquilinum in Mice Gastric Mucosa: Synergistic Effect with Helicobacter pylori Infection
Source: PLoS One. 2012 Jun 13;7(6):e38353. doi: 10.1371/journal.pone.0038353 (PMC3374793; doi:10.1371/journal.pone.0038353)
Supplement: Table S1 — Characterization of inflammation and immunohistochemistry analysis of carbohydrate antigens expression in the gastric mucosa of all mice evaluated in each experimental group. (PDF) [file pone.0038353.s002.pdf]

Table S1

	Animal	Weeks	**Inflammation** (Sidney classification)	Tn	STn	T	ST	SLe ^{a}	SLe ^{x}
**Group 1 - Control**	1.1	4	Absent	+	-	+	-	-	+
	1.2	4	Absent	+	-	+	-	-	+
	1.3	4	Absent	+	-	+	-	-	+
	1.5	7	Absent	+	-	+	-	-	+
	1.6	7	Absent	+	-	+	-	-	+
	1.7	7	Absent	+	-	+	-	-	+
	1.8	7	Absent	+	-	+	-	-	+
**Group 2 - *Pteridium aquilinum***	2.1	4	Mild	+	-	+	+	-	++
	2.2	4	Mild	+	-	+	-	-	+
	2.3	4	Mild	++	-	+	+	-	++
	2.4	4	Mild	++	-	+	+	-	++
	2.5	7	Mild	++	-	+	-	-	+
	2.6	7	Mild	++	-	+	+	-	++
	2.7	7	Mild	+	-	+	+	-	++
	2.8	7	Mild	++	-	+	+	-	++
**Group 3 - *Helicobacter pylori***	3.1	4	Moderate					-	+
	3.2	4	Moderate	+	-	+	-	-	+
	3.3	4	Mild	+	-	+	-	-	+
	3.4	4	Moderate					-	++
	3.5	7	Mild	+	-	+	-	-	+
	3.6	7	Moderate	+	-	+	-	-	++
	3.7	7	Mild						
	3.8	7	Moderate	++	-	+	-	-	++
**Group 4 - *Pteridium aquilinum* + *Helicobacter pylori***	4.1	4	Severe	++	-	+	-	-	++
	4.2	4	Severe					-	++
	4.3	4	Severe						
	4.4	4	Severe					-	++
	4.5	7	Severe	++	-	+	-	-	++
	4.6	7	Severe	++	-	+	+	-	++
	4.7	7	Severe	++	-	+	+	-	++
	4.8	7	Severe	++	-	+	+	-	++
