# Supplementary material for: Glycophenotypic Alterations Induced by Pteridium aquilinum in Mice Gastric Mucosa: Synergistic Effect with Helicobacter pylori Infection
Source: PLoS One. 2012 Jun 13;7(6):e38353. doi: 10.1371/journal.pone.0038353 (PMC3374793; doi:10.1371/journal.pone.0038353)
Supplement: Table S2 — Significantly altered genes in Pteridium aquilinum treated gastric mucosa (Group 2) in comparison with control (Group 1) from the Glyco-gene Chip array analysis. (PDF) [file pone.0038353.s003.pdf]

Table S2

Gene name	Accession no.	Fold difference	p value
		**Group 2 vs Group 1**	
**Glycan biosynthesis, modification, and degradation**			
Glycoprotein			
AHSG – Alpha-2-HS-glycoprotein	NM_013465.1	2.00	0.066924
Proteoglycan			
COL14A1 – Collagen, type XIV, ALPHA 1	uc007vse.1	1.42	0.042605
DCN – decorin	NM_007833.4	2.21	0.042605
GPC1 – glypican 1	NM_016696.4	2.22	0.025752
GPC3 – glypican 3	NM_016697.2	1.47	0.008916
GPC6 – glypican 6	NM_011821.2	1.33	0.066924
Glycan-transferase			
ST3GAL2 – ST3 beta-galactoside alpha-2,3-sialyltransferase 2	uc009nlk.1	1.42	0.091279
ST6GALNAC6 – ST6 (alpha-N-acetyl-neuraminyl-2,3-beta-galactosyl-1,3)-N-acetylgalactosaminidine alpha-2,6-sialyltransferase 6	NM_016973.2	1.33	0.025752
GALNTL4 – UDP-N-acetyl-alpha-D-galactosamine:polypeptide N-acetylgalactosaminyltransferase-like 4	NM_173739.3	1.36	0.089129
WBSCR17 – Williams-Beuren syndrome chromosome region 17 or Galnt17	NM_145218.3	1.45	0.025752
C1GALT1 – core 1 synthase, glycoprotein-N-acetylgalactosamine 3-beta-galactosyltransferase, 1	NM_052993.2	- 1.44	0.025752
DSEL – dermatan sulfate epimerase-like	NM_001081316.1	1.37	0.057607
Glycan degradation			
SULF1 – sulfatase 1	NM_172294.1	1.57	0.035948
NEU2 – sialidase 2 (cytosolic sialidase)	NM_015750.2	1.97	0.027935
GLB1 – galactosidase, beta 1	NM_009752.1	- 1.47	0.074119
Sulfotransferase			
SULT1A1 – sulfotransferase family, cytosolic,1A,phenol-preferring, member1	NM_133670.1	1.62	0.049073
**Inflammation and host immune response**			
Chemokines			
CCL21 – chemokine (C-C motif) ligand 21b (serine)	NM_011124.4	1.93	0.042605
CCRL1 – chemokine (C-C motif) receptor-like 1	NM_145700.1	1.35	0.042605
Interleukins and receptors			
IL6ST – interleukin 6 signal transducer (gp130, oncostatin M receptor)	NM_010560.2	1.47	0.052724
C-type lectin			
CD248 – CD248 molecule, endosialin	NM_054042.2	1.35	0.072165
MGL2 – macrophage galactose N-acetyl-galactosamine specific lectin 2	NM_145137.2	1.41	0.089129
**Signaling pathways and cell-cell signaling**			
Growth factors and receptors			
ANGPL2 – angiopoetin-like 2	NM_011923.4	1.67	0,035948
ANGPTL3 – angiopoetin-like 3	NM_013913.3	1.87	0,091122
NOG – noggin	NM_008711.2	1.38	0,035948
TGFB3 – transforming growth factor, beta 3	NM_009368.2	1.83	0,027935
WNT4 – wingless-type MMTV integration site family, member 4	NM_009523.1	1.81	0,079129
FZD2 – frizzled homolog 2 (Drosophila)	NM_020510.2	1.75	0,035948
FZD7 – frizzled homolog 7 (Drosophila)	NM_008057.3	2.38	0,027935
FGFR2 – fibroblast growth factor receptor 2	NM_010207.2	1.59	0,088258
FGF13 – fibroblast growth factor 13	NM_010200.2	1.68	0,042605
IGF2 – insulin-like growth factor 2 (somatomedin A)	NM_010514.2	1.55	0,056767
IGFBP4 – insulin like growth factor binding protein 4	NM_010517.3	1.94	0,042605
IGFBP5 – insulin like growth factor binding protein 5	NM_010518.2	2.36	0,035948
IGFBP6 – insulin like growth factor binding protein 6	NM_008344.2	2.\26	0,042605
BMP3 – bone morphogenetic protein 3	NM_173404.2	1,44	0,066924
FIGF – c-fos induced growth factor (vascular endothelial growth factor D)	NM_010216.1	1,45	0,056767
IHH – Indian hedgehog homolog (Drosophila)	NM_010544.2	- 1,39	0,042605
Adhesion molecule			
CD34	NM_133654.3	1,85	0,042605
PODXL2 – podocalyxin-like 2	NM_176973.3	1,30	0,066924
Nucleotide sugar			
PGM5 – Phosphoglucomutase 5	NM_175013.2	2,65	0,049073
