# Supplementary material for: Glycophenotypic Alterations Induced by Pteridium aquilinum in Mice Gastric Mucosa: Synergistic Effect with Helicobacter pylori Infection
Source: PLoS One. 2012 Jun 13;7(6):e38353. doi: 10.1371/journal.pone.0038353 (PMC3374793; doi:10.1371/journal.pone.0038353)
Supplement: Table S3 — Significantly altered genes in Pteridium aquilinum treated and Helicobacter pylori infected gastric mucosa (Group 4) in comparison with control (Group 1) from the Glyco-gene Chip array analysis. (PDF) [file pone.0038353.s004.pdf]

Table S3

Gene name	Accession no.	Fold difference	p value
		**Group 4 vs Group 1**	
**Glycan biosynthesis, modification, and degradation**			
Glycoprotein			
MUC13 – mucin 13, cell surface associated	NM_010739.2	3,36	0,046789
Proteoglycan			
CD74 – CD74 molecule, major histocompatibility complex, class II invariant chain	NM_010545.3	3,05	0,059556
Glycan-transferase			
CHST8 – carbohydrate (N-acetylgalactosamine 4-0) sulfotransferase 8	NM_175140.3	- 1,31	0,059556
B3GALT1 – UDP-Gal:betaGlcNAc beta 1,3-galactosyltransferase, polypeptide 1	NM_020283.2	- 1,36	0,054537
FUT4 – fucosyltransferase 4 (alpha (1,3) fucosyltransferase, myeloid-specific)	NM_010242.3	1,91	0,067756
Sulfotransferase			
SULT1B1 – sulfotransferase family, cytosolic, 1B, member 1	NM_019878.4	1,74	0,059556
**Inflammation and host immune response**			
Chemokines			
CCL5 – chemokine (C-C motif) ligand 5	uc007kpi.1	2,89	0,059556
CXCR6 – chemokine (C-X-C motif) receptor 6	NM_030712.4	1,63	0,066649
CXCL6 – chemokine (C-X-C motif) ligand 6 (granulocyte chemotactic protein 2)	NM_009141.2	1,60	0,054537
CCL20 – chemokin (C-C motif) ligand 20	NM_016960.1	1,64	0,067756
	uc007bsm.1	1,46	0,059556
C-type lectin			
KLRA9 – killer cell lectin-like receptor subfamily A, member 9	NM_010651.3	- 1,86	0,012733
SFTPD – surfactant protein D	NM_009160.1	2,50	0,006935
**Signaling pathways and cell-cell signaling**			
Growth factors and receptors			
FZD3 – frizzled homolog 3 (Drosophila)	NM_021458.1	- 1,36	0,054537
Adhesion molecule			
PODXL2 – podocalyxin-like 2	NM_176973.3	- 1,35	0,066649
Nucleotide sugar			
SLC35F2 – solute carrier family 35, member F2	NM_028060.3	1,90	0,054537
